# A comparative study of portable ultrasound devices in the evaluation of Atlantic bottlenose dolphin mammary gland morphology

**DOI:** 10.1098/rsbl.2025.0230

**Published:** 2025-10-15

**Authors:** María José Robles-Malagamba, Guillermo J. Sánchez-Contreras, Jan Wölfer, Mason N. Dean

**Affiliations:** ^1^ City University of Hong Kong Jockey Club College of Veterinary Medicine and Life Sciences, Hong Kong, Hong Kong; ^2^ Veterinary Medicine, The Dolphin Company, Cancun, Quintana Roo, Mexico; ^3^ Humboldt-Universitat zu Berlin Mathematisch Naturwissenschaftliche Fakultat, Berlin, Germany; ^4^ Centre for Nature-Inspired Engineering, City University of Hong Kong, Kowloon Tong, Hong Kong

**Keywords:** diagnostic imaging, ultrasonography, marine mammal research, portable ultrasound machine, handheld ultrasound scanner, veterinary medicine

## Abstract

Diagnostic ultrasound imaging is vital in human and veterinary medicine, facilitating non-invasive examinations and enhancing clinical outcomes. However, despite the increasing advances in ultrasound technology, there are few animal studies comparing scanner performance, particularly between cart-based and handheld models. We evaluated the performance of three ultrasound scanners—the cart-based Sonosite Edge II and handhelds Clarius Scanner C3 HD3 and Edge SS-H6—on mammary gland assessment of female Atlantic bottlenose dolphins (*Tursiops truncatus*) under human care. Advantages and disadvantages of each scanner for field-based measurements were assessed. Sonosite provided superior image quality but was unsuitable for performing scans in sea pens due to its weight, need of AC power and lack of waterproofing. Clarius and Edge offered greater portability, allowing examinations during adverse weather, although Wi-Fi connectivity could be hampered if mobile devices linked to the transducer connected to other networks during scanning. In showing that mammary glands increase in thickness with age, we highlight the need for consistent equipment use for organ measurements, as the variability among scanners could impact assessments and physiological interpretations (e.g. reproductive stage). This research emphasizes the importance of environmental factors and device characteristics when selecting ultrasound equipment for marine mammal studies in artificial and natural habitats.

## 1. Introduction

Ultrasonography is invaluable for veterinarians, allowing real-time, non-invasive evaluation of various organs using portable equipment [1–3]. The versatility of ultrasound enables application across diverse animal species, including small, farm, wild and aquatic animals [4–8]. Given the challenges posed by aquatic habitats and the large size of many species, ultrasonography is the preferred diagnostic imaging modality for marine mammal veterinarians [9], particularly for monitoring reproductive, abdominal and thoracic health [10–13]; however, characteristics inherent to these animals and their aquatic habitats can challenge successful assessments. Recent handheld ultrasound devices help address these issues through their enhanced portability, wireless communication and ease of use [14–16]. These innovative devices often feature lightweight, waterproof designs, integrated LED goggles and built-in Wi-Fi connectivity to tablets or smartphones, facilitating data access [16–18].

Despite increased use of handheld ultrasound devices for veterinary clinical and fieldwork, particularly improving examination efficiency and research in marine mammal medicine [19], there remains a notable lack of comparisons of ultrasound devices and their efficacy for examining diverse species. Only one study compared portable and fully equipped clinical ultrasound equipment for assessing thyroid size in Indo-Pacific bottlenose dolphins (*Tursiops aduncus*), showing that cart-based and portable units were equally effective [20]. In contrast, studies in human medicine have begun to evaluate handheld equipment [14,21] and show that while cart-based machines typically offer higher diagnostic accuracy, handheld devices can achieve comparable results in specific applications, such as cardiac and abdominal imaging [22] and situations where it is impossible to transport patients to emergency facilities [23].

The current study addressed the need for assessments of portable ultrasound devices in marine mammal medicine, drawing on data from a large ongoing project evaluating mammary gland morphology in female Atlantic bottlenose dolphins (*Tursiops truncatus*), across age and reproductive stage. As dolphins examined in that project come from multiple habitats with different available equipment, in the current study, we were able to opportunistically evaluate the performance of three ultrasound scanners (one cart-based, two handheld). Our multi-month study compared factors pertinent for veterinarians, thereby offering guidelines for optimal scanner selection for health assessments and research in managed and wild cetacean populations.

## 2. Methodology

### (a) Animals and locations

For the larger parent study, 28 healthy female Atlantic bottlenose dolphins from six Dolphin Discovery facilities in Mexico were selected for mammary gland ultrasonographic assessment. Three enclosures were 3-m-deep outdoor pools with natural seawater systems; two were sea pens with open ocean access separated by fences; and one featured sea pens separated from an adjacent marina by artificial barriers. Animals were 4−53 years old, including five sexually immature and 23 sexually mature females (electronic supplementary material, table S1). For this study, pregnant and lactating animals were excluded, to avoid mammary gland thickness fluctuations we have observed with stages of reproduction [24], leaving 18 animals. Dolphins were previously trained for medical procedures, including ultrasound examinations.

### (b) Ultrasound scanners

**Figure 1.**
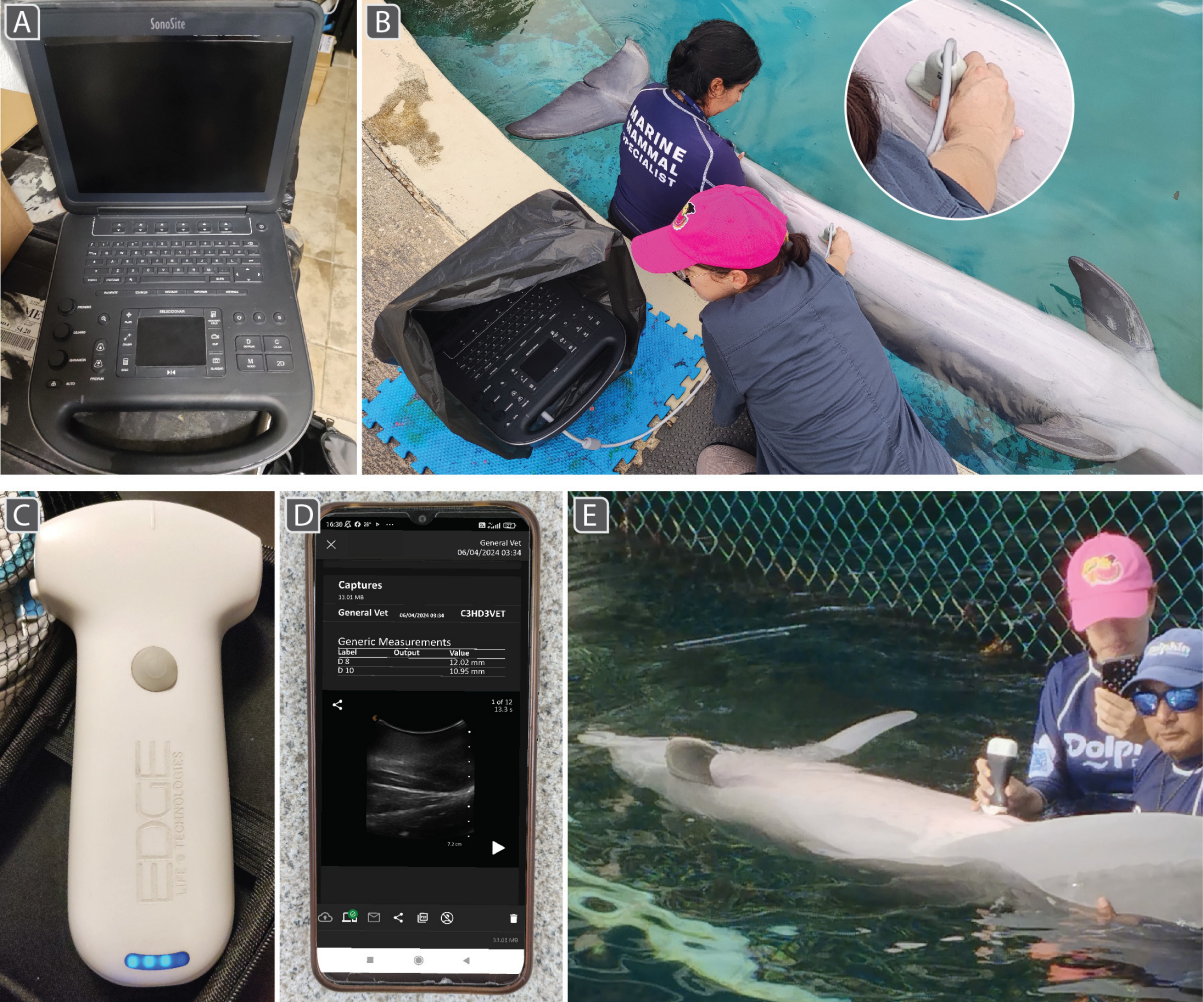
Ultrasound scanners and mammary gland examinations performed by the same operator. (A) Cart-based Sonosite, (B) used in an outdoor artificial pool and shielded from sunlight and splashes inside a box and black garbage bag. Note the trainer positioning the animal for scanning; the enlargement shows the transducer’s cord, connected to the machine. (C) Edge convex transducer; lights indicate battery level. (D) Smartphone displaying the Clarius app with mammary gland scan. (E) Exam conducted in open-water sea pen on an underwater platform. A trainer holds the dolphin, while the veterinarian performs the exam using the Clarius, consulting the smartphone app.

The ultrasound scanners used depended on those available and maintained by in-house veterinarians at each facility. All ultrasound examinations were conducted by the same veterinarian. For animals located in the closed-pool systems and the marina, examinations were conducted using a cart-based Sonosite Edge II (Sonosite Inc., Bothell, WA, USA) (figure 1A,B) equipped with a convex transducer (model rC60xi). A handheld wireless Clarius Scanner C3 HD3 (Clarius Mobile Health, Vancouver, Canada) (figure 1E), connecting through built-in Wi-Fi to a mobile device for real-time image visualization and storage (figure 1D), was used for animals in Cozumel and sometimes Isla Mujeres. A handheld wireless Edge SS−6H (Edge Life Technologies, Miami, FL, USA), also a convex transducer connecting through Wi-Fi to a mobile device (figure 1C), was used for most examinations in Isla Mujeres. In this study, no animal was scanned with all three ultrasound units (electronic supplementary material, table S1). Table 1 compares scanner specifications; we refer to the scanners as Sonosite, Clarius and Edge, respectively.

**Table 1.** Ultrasound scanner specifications.

technical details	Sonosite edge II	Clarius C3 HD3	Edge SS−6H
length (cm)	32.6	14.6	14.5
width (cm)	30.7	7.6	7
thickness (cm)	—	3.2	1
weight (kg)	4.18 with battery	0.308	0.180
height (cm)	6.4	—	—
handheld	no	yes	yes
built-in battery	yes		
Wi-Fi connection	no	yes	yes
grey scale	256 shades, range adjustable by operator		
probe frequency range	2−5 MHz: preset in unit, cannot be modified; strong penetration with mid-range resolution; includes real-time image optimization	2−6 MHz: can be adjusted; AI-enhanced imaging and software-based beamforming	3.2−5.0 MHz: can be adjusted; enhanced gradient sharpness, contrast and edge processing
gain settings	adjusted by operator		
scan depth	≤30 cm	≤40 cm	9−30 cm
waterproof	no	1 m for 30 min	yes
exam types (manufacturer presets that exist in the unit; however, all three scanners in this project can conceivably be used for any standard anatomical application)	abdominal gynaecological musculoskeletal neural obstetrical	abdominal cardiac cephalic (adult) fetal gynaecological intra-operative musculoskeletal paediatric peripheral vessel urological	abdominal gynaecological hepatic pulmonary renal urological
storage	16 GB internal flash memory	8 GB of storage (on-board); 1 GB of memory	depends on mobile device storage capacity
images saved as	JPEG	DICOM, JPEG, PNG, BMP	DICOM, JPEG, PNG
videos saved as	MP4, DICOM	AVI, DICOM	MP4, DICOM
battery duration	2 h scanning time, depending on imaging mode/display brightness	45 min scanning time; from empty battery approx. 1.5 h to full charge	2.5 h continuous scan; wireless charging, 2 h to full charge
references	[25,26]	[27,28]	[29]

### (c) Ultrasonographic examinations

Ultrasound scans of right and left glands were performed over a five-month period, from November 2023 to April 2024. Animals were scanned weekly at each habitat for approximately 10 min each (figure 1B,D), contingent upon weather conditions and trainer/animal availability. In artificial habitats, exams were performed at the pool edge (figure 1B). In sea pens, exams were performed by the veterinarian either from the docks or standing on underwater platforms (figure 1D), with animals positioned and stabilized in dorsal recumbency by their trainer. To visualize mammary glands, the transducer was moved cranially from the cranial genital slit edge until the gland parenchyma was identified. The longitudinal aspect of the right mammary gland was scanned first, from caudal to cranial border, then the probe rotated 90° to scan the gland transversely from cranial to caudal. The animal was rotated 180° to scan the left mammary gland.

### (d) Imaging and mammary gland measurements

**Figure 2.**
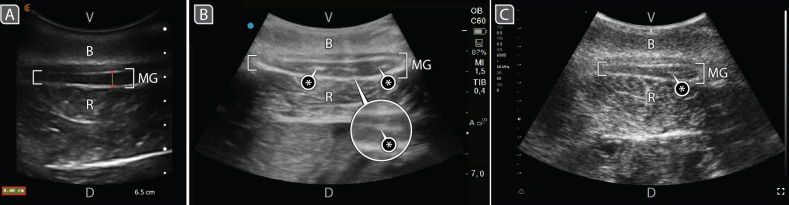
B-mode ultrasound images of the right mammary gland of one dolphin taken with three different scanners on different days. MG, mammary gland; V/D, ventral/dorsal aspects of the animal, respectively; B, blubber; R, rectus abdominis muscle. Asterisks indicate closed mammary ducts (hyperechoic lines). (A) Clarius, (B) Sonosite and (C) Edge scanners. Ultrasound wave scattering produces the speckle artefact (textured appearance) in image (C). The Clarius logo and blue dot (top left of images (A) and (B), respectively) indicate the direction the probe is pointing. Scan depth = 7.5 cm (A), 7 cm (B), 9 cm (C).

Ultrasound image quality was optimized differentially, depending on the unit. With the Sonosite, manual frequency adjustment was not possible; instead, predefined settings were used (i.e ‘Penetration’, ‘Resolution’) to optimize image quality. In contrast, Clarius and Edge units allowed for direct frequency modulation. A high frequency was selected for these units (e.g. 5 MHz), as mammary glands are superficial in the ventral abdomen (figure 2); higher frequencies enhance resolution, thereby improving the differentiation of closely spaced anatomical structures. The Sonosite allowed for manual gain adjustments, which we calibrated to approximately 63 dB. The Clarius featured automatic gain adjustment for real-time image quality optimization, ensuring consistent performance across scanning conditions. Edge gain settings were adjusted from 70 to 100 dB, depending on individual animal characteristics (e.g. blubber thickness). Standard depth penetration for ultrasound scanners was set at 8 cm and the focal zone was adjusted ± 3 cm, depending on mammary gland size.

During the first scanning month, images were captured of the thickest portion of both mammary glands, longitudinal and transverse aspects in all animals, through real-time B-mode trans-abdominal ultrasound. In the second month, the method was refined and images captured instead from videos of both mammary gland aspects. Depending on the scanner, images were saved either in DICOM, JPEG or PNG formats, while videos were saved in AVI or MP4 formats (table 1). Videos were converted to 50 images using the Free Video-to-JPG Converter (Media Freeware). Mammary gland thickness from all subjects was measured from the derived cross-sectional gland images using the distance function in the MicroDicom DICOM viewer app (figure 2A).

### (e) Scanner evaluation

Scanners were evaluated qualitatively with regard to imaging, performance and ease of use. The analysis was conducted by the veterinarian responsible for all ultrasound examinations, who possesses prior expertise in analysing mammary gland scans in dolphins (e.g. *Tursiops aduncus*). Image quality was assessed according to clarity of anatomical features (see §3 Results) and in an unblinded manner, as the display characteristics of each scanner were too distinctive and recognizable. However, to reduce the inherent degree of bias from reliance on a sole image quality evaluator, data from the different scanners was evaluated in random order over the evaluation period (i.e., images from one scanner type were not analysed as a batch). For the qualitative image analysis, both DICOM and JPEG images were used and while comparison of image quality in two different formats may carry some limitations, this was unavoidable as the image format was dictated by the manufacturer of the scanners as a factory setting. For consistency, only the first author assessed the images and only the MicroDicom viewer was used to conduct thickness measurements.

For statistical analyses, R version 4.4.3 [30] and packages nlme [31] and emmeans [32] were used. To test the effect of scanner model on gland thickness, we used a stepwise statistical model-building procedure [33] to obtain a model meeting statistical assumptions while controlling for factors significantly affecting gland thickness. To obtain a final model that was as simple as possible but as complex as necessary for reliable inferences, factors (i.e. independent variables) and assumptions about the covariance structure of model errors were altered in a stepwise manner. With each change (i.e. a factor removal), the more complex model was compared with the simpler model (nested within the complex model) using a likelihood ratio test. If the *p*-value was small enough (alpha approx. 0.05), the more complex model was retained, otherwise, the simpler model was preferred. These decisions were further informed by graphical diagnostics of the model residuals, as well as model comparisons using Akaike and Bayesian information criteria (AIC and BIC) [34]. Both indices weigh model likelihood against model complexity, with BIC penalizing complexity more, thus tending to prefer simpler models. Model comparison was performed only with models fitted using the same estimation method. We used maximum likelihood estimation to compare models with differing fixed effects and restricted maximum likelihood otherwise. We considered age and reproductive stage as important independent variables *a priori*, but not body length, body side (left or right gland), location (sea pen or pool) and numbers of prior pregnancies, since only age and reproductive stage were found to have a significant effect on gland thickness [24]. See electronic supplementary material for the intermediate results of the model building procedure.

The final statistical model contained age and scanner model as fixed effects. To examine the effect of age we used the *intervals* function to obtain 95% confidence intervals (CI) and *summary* function to obtain the *p*‐value. For the effect of scanner model, the *contrast* function from the R package emmeans was used to obtain 95% CIs and *p*-values. The Tukey correction was used to adjust these values for multiple group comparisons.

## 3. Results

### (a) General evaluation

Scanners were compared according to relevant factors for successful mammary gland examination in marine mammals (table 2): ability to differentiate mammary gland structures (i.e. image quality), ease of use and portability, battery life and tendency for overheating and Wi-Fi connectivity. We analyse specific advantages and disadvantages in §4 Discussion.

**Table 2.** Qualitative evaluation of ultrasound scanners.

	Sonosite Edge II	Clarius C3 HD3	Edge SS−6H
ability to differentiate mammary gland structures	good to excellent: clear margin definition and contrast; sharp tissue interfaces and minimal noise in the image (figure 2A)	good: acceptable image contrast and soft tissue planes easily differentiated. High contrast due to onboard postprocessing. Gland borders are clear but less crisp than in the Sonosite Edge II (figure 2B)	moderate: grainier image (speckle reduction settings difficult to adjust with one operator). Gland margins and deeper structures less defined; loss of resolution in axial and lateral planes (figure 2C)
ease of use	keyboard easy to navigate, rapid access to functions like gain adjustment, for single operator use. Transducer cord short for exams performed from high docks	App easy to use, efficient exams with single operator, as transducer has start/stop scan button; difficult to navigate on tablet for underwater platform scans	MicroVue app not as easy to adjust image quality settings and installation in Android smartphones; an iPad was used, challenging to manoeuvre for single operators
portability	heavy to carry for long periods of time, needed to be placed in the ground/platform during exams; not ideal for scanning animals in sea pens	excellent portability when scanning in sea pens, easy movement of transducer and operator if the animal moved or a wave reached the area	excellent portability when scanning in sea pens, easy movement of transducer and operator if the animal moved or a wave reached the area
battery life	if more than three animals to scan, battery charging needed, challenging in facilities with limited access to AC power plugs	scanner left to charge overnight, allowing scans of more than three animals in a row; charging battery between scans, time-consuming	scanner was used in facility with 11 animals to scan a day; needed to be charged more frequently than the other scanners
overheating	concerning in presence of high temperatures and lack of shade, covered for outdoor use	internal battery can overheat in high temperatures, important to protect it from prolonged sunlight	did not overheat when used for prolonged time, caution was taken not to overexpose it to direct sunlight
Wi-Fi connectivity and file saving/transfer	no ability to connect to mobile devices (e.g. through Wi-Fi); data written to USB, resulting in prolonged image saving times	rapid image storage and sharing with built-in Wi-Fi, but other networks could interrupt scanning by connecting to the mobile device used	rapid image storage and sharing with built-in Wi-Fi, but other networks could interrupt scanning by connecting to the mobile device used

### (b) Quantitative analysis

Twelve individuals were scanned using Sonosite, seven by Clarius and six by Edge (electronic supplementary material, table S1, figure S1). Individual dolphins were measured for variable numbers of weeks throughout the scanning period due to logistical aspects of working at different facilities (electronic supplementary material, tables S3–S6). As a result, 18−42 measurements were taken per individual from the Sonosite, 2−7 from the Clarius, and 6−10 from the Edge.

**Figure 3.**
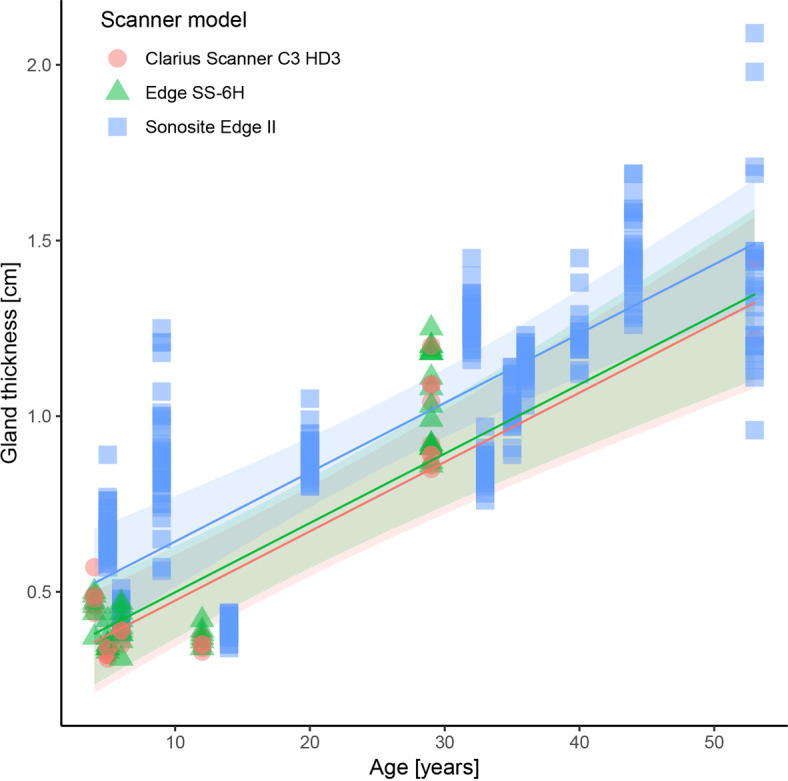
Regressions indicating expected values for gland thickness as a function of age and scanner model; shaded area = 95% confidence intervals.

Age (electronic supplementary material, table S1) had a significant effect on gland thickness (*p* < 0.001), which increased on average 0.02 cm (CI: 0.014, 0.026) per year (table 3, figure 3). Differences between Sonosite and each of the handheld models were larger than the difference between Edge and Clarius. For a given age, measurements with the Edge resulted in a gland thickness on average 0.024 cm (CI: 0.001, 0.046) larger than the Clarius (*p* = 0.039; table 3; figure 3). In turn, measurements with the Sonosite resulted in a gland thickness on average 0.168 cm (CI: −0.019, 0.356) larger than the Clarius (*p* = 0.089) and 0.145 cm (CI: −0.043, 0.332) larger than the Edge (*p* = 0.168; table 3; figure 3). Hence, scanner types show either small, significant differences (i.e. between Edge and Clarius), or larger, but statistically insignificant differences (i.e. Sonosite relative to the others). This indication of a limited effect of scanner type reflects model selection analysis results, in which the BIC preferred the model only including age over that including both age and scanner type (electronic supplementary material, table S2). Dolphins nos. 1 and 5 displayed strong fluctuations (>0.6 cm) in gland thickness over the measurement period (electronic supplementary material, figure S1). In dolphins nos. 5 and 18, gland thickness appeared to increase over time despite animals being neither pregnant nor lactating (electronic supplementary material, figure S1).

**Table 3.** Statistical results of the final model used for inference, including scanner and age as fixed effects.

scanner model							
contrast	estimate (cm)	s.e.	d.f.	lower CL	upper CL	*t*-value	*p*‐value
Edge - Clarius	0.024	0.01	426	0.001	0.046	2.452	0.039
Sonosite - Clarius	0.168	0.08	426	−0.019	0.356	2.108	0.089
Sonosite - Edge	0.145	0.08	426	−0.043	0.332	1.809	0.168

age							
effect	estimate (cm year^−1^)	s.e.	d.f.	lower CL	upper CL	*t*-value	*p*‐value
slope	0.02	0.003	16	0.014	0.026	6.82	<0.001

Contrast: difference between mean thickness measurements of the two scanner models mentioned, for a given age; SE: standard error; d.f.: degrees of freedom; CL: confidence limit.

## 4. Discussion

Most studies comparing ultrasound scanner performance have focused on accuracy and reproducibility of organ measurements and the assessment of particular body systems, rather than evaluating ease of use and scanner performance in fieldwork settings. These studies have largely centred on improving diagnostic effectiveness in controlled human clinical environments [14,20–22]. In veterinary medicine, the diversity of animal species and their habitats demand more varied and flexible ultrasound scanning protocols. Yet, comparisons of diagnostic imaging options tailored to disparate veterinary settings remain rare: one of the only studies involved a qualitative comparison of six high-resolution ultrasound transducers evaluating ovarian follicle development in adult female common marmosets (*Callithrix jacchus*) at penetration depths relevant for preclinical studies [35]. This qualitative analysis was juxtaposed with quantitative evaluation of nine ultrasound transducers based on image quality (relating ultrasound beam penetration to beam width; [36]). Significant variation among transducer types was found in factors important for diagnostic imaging (depth of field, image quality and resolution), emphasizing the value in establishing protocols for rigorous evaluations of imaging performance and—as some scanners proved more effective for *in vivo* imaging—in comparing them for specific clinical applications.

Ultrasonographic research of mammary glands in cetaceans is also scarce, including just two works: a cross-sectional study of how left mammary gland thickness varies with reproductive stage in 28 live-captured finless porpoises (*Neophocaena asiaeorientalis sunameri*) [37] using a cart-based LOGIQ Book XP unit (General Electric Co., Schenectady, NY, USA) and a longitudinal study by cart-based ultrasound (Sonosite Titan; Sonosite Inc., Bothell, WA, USA), examining mammary glands of three Atlantic bottlenose dolphins in pools. The latter study analysed urinary prolactin concentration and its correlation with mammary gland thickness at different reproductive stages [38]. In comparison, our study provides a broader context, involving mammary gland examinations in a longitudinal study of a large sample of Atlantic bottlenose dolphins inhabiting three very distinct habitats and comparing different ultrasound scanners. The logistical difficulties we faced in our larger study (e.g. time, weather and scanner constraints), which resulted in inconsistent scanning conditions and timelines, allowed a unique evaluation of ultrasound performance in challenging fieldwork conditions faced by marine mammal veterinarians. Moreover, this is the first quantitative evaluation of bottlenose dolphin mammary glands with handheld ultrasound units.

The scanners used presented particular advantages and disadvantages for marine animal work, especially for imaging cetacean mammary glands. With regard to resolving mammary gland structures, Sonosite and Clarius image quality, for example, was superior to that of Edge, the latter presenting speckle artefacts in all scans (figure 2C); with all three units, however, mammary gland borders, could be well-identified for thickness measurements (figure 2). In terms of diagnostic value, the Sonosite was preferred for the evaluation of fine structures due to its clear margin definition and contrast, while the Clarius offered adequate detail for soft-tissue assessment (e.g. resolving gland ducts within the parenchyma; figure 2B,C). However, the Edge provided less clarity and contrast, hampering diagnostic confidence when evaluating detailed structures.

Although our quantitative results demonstrated a consistent trend of mammary gland thickening with age (0.02 cm year^−1^) in the non-lactating/non-pregnant animals in this study, Sonosite measurements of mammary glands were consistently approximately 2 mm thicker than those from the other scanners (table 3, figure 3). As this represents approximately 10× our estimated annual gland thickness increase and since we have shown that mammary glands also thicken during pregnancy and lactation [24], this measurement difference indicates potential for misrepresentation of animal age/reproductive stage from gland measurements, especially if switching between scanners (figure 3, electronic supplementary material, S1). However, with *p*-values for pairwise comparisons ranging from 0.039 to 0.168 (table 3) and our comparative trends among the three scanners largely based on younger individuals (i.e. <12 years old; figure 3), more data are needed to verify this interpretation.

Qualitative evaluations of scanner function and suitability (table 2), illustrate key considerations for field-based ultrasound on aquatic animals. For example, sunlight posed a significant risk for equipment overheating. To mitigate this, in facilities with more than five dolphins to evaluate, scanning was spread over the day, with transducers moved to shaded areas between examinations. Notably, the size and battery type of handheld devices allowed for longer scanning durations without shade, compared with the cart-based scanner. However, the Clarius would automatically shut down within 30 s if the transducer became too warm, which could disrupt scanning procedures. Regarding image visualization in well-lit environments, the Sonosite screen, despite having very good resolution, needed shade for clear image visualization and analysis. Conversely, when both handheld scanners were connected to smartphones, image visualization was very clear even in sunlight, due to the high brightness of smartphone screens. However, when the transducers were connected to tablets, screen glare from their waterproof covers hindered clear visualization.

Of particular relevance for cetacean work, compared with both handheld devices, the Sonosite was not waterproof; it was therefore seldom used in sea pen facilities to avoid the risk of seawater damage and electrocution. The short transducer cord (1.7 m; figure 1B) posed additional challenges, as it could not reach animals if scanned in pens with high docks and underwater platforms. In contrast, the portability of handheld units allowed efficient examinations in sea pens, even during high tides and poor weather, without equipment or electrocution risks. Notably, after prolonged use, the rubberized covering of the *start/stop* button on the Edge (figure 1C) began to degrade; water eventually entered the unit’s interior through perforations around this button, and the equipment had to be repaired. Portability, however, did not always equate to handleability for measurements in challenging conditions: it was difficult to adjust image settings during underwater platform examinations, if animals were scanned without the help of another veterinarian, as this required holding the scanner with one hand and the visualization device with the other. This was especially demanding with tablets (larger screen to control with one hand) or during unfavourable weather conditions (e.g. rain, high tides). We therefore argue that team composition is an unappreciated importance in scanner choice (i.e. whether others will assist in procedures).

All scanners were limited to some degree by the need for connectivity (e.g. for power, internet). For example, if ultrasound exams were lengthy, the Sonosite (battery life approximately 2 h) needed to be connected to AC power through long extension cords, as power plugs were located far from pools for safety; this was particularly challenging working in sea pens, which can be larger than dolphinarium pools [39]. On the other hand, although the Clarius and Edge are equipped with chargers, these are not wireless; therefore, with low battery, transducers had to be physically returned to charging stations. Concomitantly, as these units rely on their integrated wireless network to save scan data, other nearby networks could interfere with the mobile device connection to the transducer if it was set to connect automatically to any of these networks. This situation would cause prolonged scanning times, as the mobile device would have to be paired again with the transducer and the procedure would need to be repeated from the start. However, this could be avoided by ensuring that the mobile device in use was not set to ‘auto-connect’ to any other Wi-Fi networks prior to the ultrasonographic exam. Moreover, the probes of handheld ultrasound units must remain above water for Wi-Fi transmission, and therefore cannot be fully submerged. In contrast, cart-based units like the Sonosite Edge maintain ultrasound signals even when transducers are submerged. This distinction is crucial when performing dolphin ultrasonography, either ‘poolside’ or in sea pens during high tides when waves may fully submerge the probe.

Finally, the aquatic nature of dolphins created unavoidable movements during ultrasound examinations, which although minimized by trainers, made obtaining successful images challenging. Moreover, all medical procedures were voluntary, meaning the dolphins could choose to swim away from the scanner at any time. The handheld scanners offered significant advantages in this context, providing ease of movement throughout examinations even if the dolphin shifted from its original position. In contrast, the Sonosite, being heavier (>4 kg with battery; table 1) and less portable, was cumbersome to reposition when dolphins moved further from the operator. It is worth noting, however, that mammary glands of this species are superficial (< 5 cm from the caudal abdominal wall; figure 2), favourable for rapid ultrasound examinations with either cart-based or handheld scanners.

In conclusion, this opportunistic analysis provided one of the first comparative evaluations of portable cart-based and handheld ultrasound scanners in bottlenose dolphins, and the first to evaluate cetacean mammary glands with handheld units for research purposes. Our evaluation suggests that while Sonosite delivers good image quality, it is less suitable for longitudinal studies involving marine mammals, especially those in sea pens where access to AC power is limited. In contrast, Clarius and Edge offer greater portability and accessibility in challenging habitats and weather conditions, although operators need to be careful to set their mobile device to only connect to the transducer’s network and to ensure that the probe is not completely submerged when performing scans.

Our results advise against switching between scanners when precise organ measurements are required, as variability introduced by different machines could significantly affect results. The inter-scanner variability suggested by our dataset, however, would benefit from further validation, especially for older animals (> 30 years old). These results highlight the need to carefully consider environmental and team conditions and the specific features of ultrasound devices when selecting scanning equipment for field applications and *in vivo* measurements for marine mammal studies, while also offering guidelines for downstream improvements to ultrasound scanners and accessories (e.g. sunshades, battery packs).

## Data Availability

Data in this study are currently being analysed for another manuscript; data specific for this study are available from Figshare where a description of the dataset is provided along with the raw data [40].
